# Interferon-*γ* Stimulates Monocyte Chemotactic Protein-1 Expression by Monocytes

**DOI:** 10.1155/S0962935194000050

**Published:** 1994

**Authors:** J. M. Liebler, S. L. Kunkel, R. M. Allen, M. D. Burdick, R. M. Strieter

**Affiliations:** 1 Department of Pathology University of Michigan Medical School Ann Arbor Michigan USA; 2 Department of Internal Medicine University of Michigan Medical School Ann Arbor Michigan USA; 3 Department of Medicine Oregon Health Sciences University 3181 S.W. Sam Jackson Park Road Portland OR 97201-3098 USA

## Abstract

Monocyte chemotactic protein (MCP-1) is a specific monocyte chemoattractant and activating factor produced by both immune cells (mononuclear phagocytes and lymphocytes) and non-immune cells (parenchymal and stromal cells). In order to define the conditions under which human monocytes express MCP-1, monocytes were exposed to IFN-*γ*, IL- l*β*, TNF-*α*, IL-4 or PHA under serum free conditions. There was no significant MCP-1 production by monocytes following exposure to IL-l*β*, TNF-*α* or IL-4. In contrast, stimulation with IFN-*γ* resulted in a dose dependent increase in MCP-1 protein and mRNA expression. Simultaneous stimulation with IFN-*γ* and IL-1*β* or TNF-*α* resulted in no further increase in MCP-1 production. It is concluded that IFN-*γ*, primarily a product of T_H_1 T lymphocytes, stimulates the expression of MCP-1 by monocytes.


Mediators of Inflammation 3, 27–31 (1994)

### Interferon-$\gamma$ stimulates monocyte chemotactic protein-1 expression by monocytes

J. M. Liebler,[3,CA] S. L. Kunkel,[1] R. M. Allen,[1] M. D. Burdick[1] and R. M. Strieter[2]

[1]Departments of Pathology and [2]Internal Medicine, University of Michigan Medical School, Ann Arbor, Michigan, USA; [3]Department of Medicine, Oregon Health Sciences University, 3181 S.W. Sam Jackson Park Road, Portland, OR 97201-3098, USA

[CA] Corresponding Author

**MONOCYTE chemotactic protein (MCP-1) is a specific monocyte chemoattractant and activating factor produced by both immune cells (mononuclear phagocytes and lymphocytes) and non-immune cells (parenchymal and stromal cells). In order to define the conditions under which human monocytes express MCP-1, monocytes were exposed to IFN-$\gamma$, IL- 1$\beta$, TNF-$\alpha$, IL-4 or PHA under serum free conditions. There was no significant MCP-1 production by monocytes following exposure to IL-1$\beta$, TNF-$\alpha$ or IL-4. In contrast, stimulation with IFN-$\gamma$ resulted in a dose dependent increase in MCP-1 protein and mRNA expression. Simultaneous stimulation with IFN-$\gamma$ and IL- 1$\beta$ or TNF-$\alpha$ resulted in no further increase in MCP-1 production. It is concluded that IFN-$\gamma$, primarily a product of $T_H1$ T lymphocytes, stimulates the expression of MCP-1 by monocytes.**

**Key words:** Chemotaxins, Cytokines, Interferon-$\gamma$, Interleukin-1$\beta$, Interleukin 4, Monocytes, Monocyte chemotactic factor-1, Phytohaemagglutinin, Tumour necrosis factor-$\alpha$

### Introduction

Monocytes are suspected of playing a key role in the development and maintenance of many acute and chronic inflammatory disease states. The recruitment of monocytes from the peripheral blood to a site of inflammation depends upon the generation of chemotactic factors by both immune cells (mononuclear phagocytes and lymphocytes) and non-immune cells (parenchymal and stromal cells). Amplification of the immune response may occur as monocytes elaborate chemotactic factors which recruit additional monocytes to the site of inflammation. Monocyte chemotactic protein-1 (MCP-1) is a potent chemoattractant and activating factor that is specific for monocytes and may play an important role in the pathogenesis of a variety of monocyte dependent disease states including atherosclerosis,[1] rheumatoid arthritis[2] and idiopathic pulmonary fibrosis.[3] Although monocytes have previously been shown to express both peptide and mRNA for MCP-1, the conditions under which they do so are controversial.[4,5] To date, *in vitro* studies have not consistently demonstrated that monocytes cultured in the absence of serum produce MCP-1 in response to stimuli that might be present near an inflammatory lesion.

In this study, the authors investigated the ability of adherence purified peripheral blood monocytes cultured in serum free media to produce MCP-1 in response to several stimuli known to activate monocytes—interleukin-1$\beta$ (IL-1$\beta$), tumour necrosis factor-$\alpha$ (TNF-$\alpha$) and interferon-$\gamma$ (IFN-$\gamma$). It was also of interest to determine whether interleukin 4 (IL-4), a product of T-lymphocytes with both pro- and anti-inflammatory properties, would stimulate monocytes to produce MCP-1. The results were compared with those obtained by exposing monocytes to a known inducer of monocyte derived MCP-1, the plant lectin, phytohaemagglutinin (PHA).[6] Although stimulation with IL-1$\beta$, TNF-$\alpha$ or IL-4 did not result in the production of monocyte derived MCP-1, it was found that exposure to IFN-$\gamma$ resulted in a dose dependent increase in the expression of MCP-1 protein and mRNA. Simultaneous stimulation with IFN-$\gamma$ and IL-1$\beta$ or TNF-$\alpha$ resulted in no further increase in MCP-1 production. These findings indicate that IFN-$\gamma$, primarily a product of the $T_H1$ subset of T lymphocytes, stimulates the expression of MCP-1 by monocytes.

### Materials and Methods

*Reagents:* Human recombinant IFN-$\gamma$ was obtained from Genzyme Corporation (Cambridge, MA, USA) and used at a concentration of $10^5$ units/ml. Phytohaemagglutinin (PHA-P; Sigma) was dis-

© 1994 Rapid Communications of Oxford Ltd solved in complete media prior to use. Human recombinant IL-1$\beta$ with a specific activity of 30 units/ng was a gift from the Upjohn Co. Human recombinant TNF-$\alpha$ with a specific activity of 22 units/ng was kindly provided by Genentech (San Francisco, CA, USA). Human recombinant IL-4 was purchased from R&D Systems (Minneapolis, MN, USA). Complete media consisted of RPMI 1640 (Whitaker Biomedical Products, Whitaker, CA, USA), without serum, with 1 mM glutamine, 25 mM HEPES, 100 U penicillin and 100 $\mu$g streptomycin/ml.

*Cell preparation:* Heparinized venous blood was obtained from healthy volunteers and mixed 1:1 with 0.9% saline. Mononuclear cells were isolated by Ficoll–Hypaque (Pharmacia) density gradient centrifugation and adherence purified, resulting in a cell population that was >90% monocytes. Cells were plated in either 35 mm or 100 mm culture plates at a final concentration of $1 \times 10^6$ cells/ml complete media. Following stimulation, mononuclear cells and supernatants were harvested at predetermined times. Cell-free supernatants were collected and spun at 2000 rpm for 10 min. Cell pellets were extracted for total RNA.

*Northern blot analysis:* Total cellular RNA from mononuclear cells was isolated using a modification of the methods of Chirgwin *et al.*[7] and Jonas *et al.*[8] Briefly, mononuclear cell pellets were overlaid with 3 ml of a solution consisting of 25 mM Tris (pH 8.0) 4.2 M guanidine isothiocyanate, 0.5% Sarkosyl, and 0.1 M 2-mercaptoethanol. After homogenization, the above suspension was added to an equal volume of 100 mM Tris (pH 8.0) containing 10 mM EDTA and 1.0% SDS. The mixture was then extracted with chloroform–phenol and chloroform–isoamyl alcohol. The RNA was precipitated with alcohol and the pellet dissolved in DEPC water. RNA was separated by Northern blot analysis using formaldehyde/1% agarose gels, transblotted to nitrocellulose, baked, prehybridized, and hybridized with a $^{32}$P 5′-end labelled oligonucleotide probe. The 30-mer oligonucleotide probe was synthesized using published cDNA sequence for human-derived MCP-1.[9] The MCP-1 probe was complementary to nucleotides 256–285 and had the sequence 5′–TTG–GGT–TTG–CTT–GTC–CAG–GTG–GTC–CAT–GGA–3′. Blots were washed and autoradiographs were quantified using a MacIntosh IIfx computer containing an Image Capture 1000 Frame Grabber (Scion Corp., Walkersville, MD, USA) and image 1.40 software (NIH Public Software, Bethesda, MD). Equivalent amounts of total RNA/gel were assessed by monitoring 28S and 18S rRNA.

*MCP-1 ELISA:* Antigenic MCP-1 was quantified using a modification of a double ligand method as described previously.[10] Flat-bottomed 96-well microtitre plates (Nunc Immuno-Plate I 96-F) were coated with 50 $\mu$l/well of rabbit anti-MCP-1 (1 ng/$\mu$l in 0.6 M NaCl, 0.26 M $H_3BO_4$, and 0.08 N NaOH, pH 9.6) for 16 h at 4°C and then washed with phosphate buffered saline (PBS), pH 7.5, 0.05% Tween-20 (wash buffer). Microtitre plate nonspecific binding sites were blocked with 2% BSA in PBS and incubated for 90 min at 37°C. Supernatants (undiluted or 1:10 dilution) or standards were added and incubated for 1 h at 37°C. Plates were washed four times and 50 $\mu$l/well of biotinylated rabbit anti-MCP-1 added, and incubated for 30 min at 37°C. Plates were washed four times, streptavidin–peroxidase conjugate (Bio-Rad Laboratories, McLean, VA, USA) added and then incubated for 30 min at 37°C. Plates were washed four times and chromogen substrate (Bio-Rad) added. The plates were incubated at room temperature to the desired extinction, and the reaction terminated with 50 $\mu$l/well of 3 M $H_2SO_4$ solution. Plates were read at 490 nm in an ELISA reader. Standards were 1/2 log dilutions of rMCP-1 (100 ng to 1 pg/ml). MCP-1 ELISA detected specific MCP-1 concentrations in a linear fashion greater than 50 pg/ml and did not cross-react with TNF-$\alpha$, IL-1$\beta$, IL-2, IL-4, IL-6, IL-8, INF-$\gamma$, ENA-78, MIP-1$\alpha$, MIP-1$\beta$, IP-10, NAP-2, RANTES, MCP-2 or MCP-3.

*Statistical analysis:* Data were analysed by a MacIntosh II computer using the Statview II statistical software package (Abacus Concepts, Inc., Berkeley, CA, USA) and expressed as means ± S.E.M. Data that appeared statistically significant were compared by Student's *t*-test and considered significant if $p$ values were $<0.05$.

### Results

*Monocyte derived antigenic MCP-1 production:* It was of interest to learn what types of stimuli resulted in the production of antigenic MCP-1 by Ficoll isolated, adherence purified peripheral blood monocytes cultured under serum free conditions. Monocytes were exposed to either PHA (10 $\mu$g/ml), IL-1$\beta$ (2.0 ng/ml), TNF-$\alpha$ (2.0 ng/ml) or IFN-$\gamma$ (10 000 units/ml) overnight. Cell-free supernatants were examined for the presence of antigenic MCP-1 using a specific MCP-1 ELISA. As shown in Fig. 1, unstimulated monocytes and monocytes stimulated with IL-1$\beta$ or TNF-$\alpha$, did not produce antigenic MCP-1. Similarly, monocytes failed to produce MCP-1 following exposure to IL-4 (10 ng/ml, $n = 4$, data not shown). Monocytes incubated in the presence of IFN-$\gamma$ produced

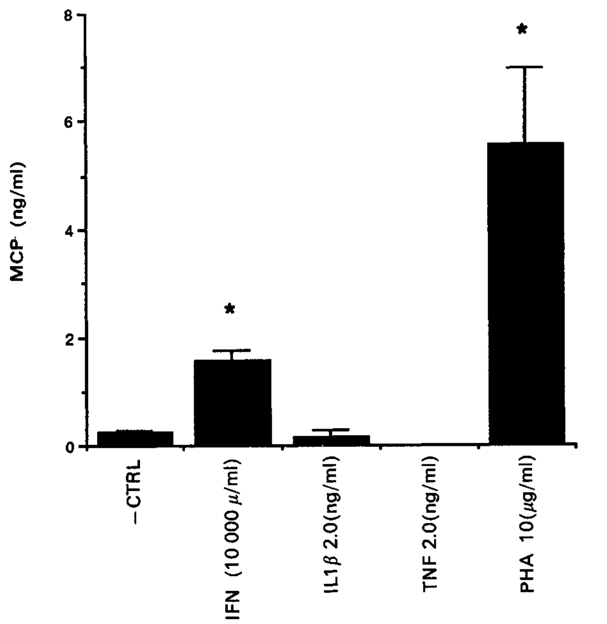

FIG. 1. Monocyte derived antigenic MCP-1 expression post-stimulation by PHA, IL-1β, TNF-α, IFN-γ as determined by ELISA. *Indicates $p < 0.05$ compared with control monocytes at comparable time point (experimental $n = 7$).

1.56 ± 0.21 ng/ml MCP-1, which was significantly increased compared with unstimulated monocytes. By comparison, monocytes stimulated with the plant lectin, PHA, produced 10.78 ± 1.48 ng/ml antigenic MCP-1, which was seven-fold greater than that generated by IFN-γ.

*Dose dependent monocyte derived antigenic MCP-1 production:* Because IL-1β and TNF-α are important components of the inflammatory response, an attempt was made to determine whether co-stimulation with IFN-γ and either IL-1β or TNF-α affected MCP-1 production by monocytes. Monocytes were exposed to varying doses of IFN-γ (1 to 10 000 units/ml) alone or in combination with either IL-1β (2 ng/ml) or TNF-α (2 ng/ml). MCP-1 production increased in a dose dependent fashion following stimulation with IFN-γ alone (Fig. 2). There was no significant change in the amount of antigenic MCP-1 generated from these cells when they were incubated with IFN-γ and IL-1β or TNF-α compared with IFN-γ alone.

*Expression of monocyte derived MCP-1 mRNA:* Northern blot analysis was performed on mRNA obtained from monocytes stimulated with varying concentrations of IFN-γ. As shown in Fig. 3, there was a dose dependent increase in MCP-1 mRNA following stimulation with IFN-γ (10 units/ml to 10 000 units/ml). There was no detectable increase in MCP-1 mRNA following exposure to the lowest dose of IFN-γ tested, 10 units/ml. Of interest was the finding that even at the maximum dose of IFN-γ used (10 000 unit/ml), the amount of steady-state mRNA was only 60% of that generated by

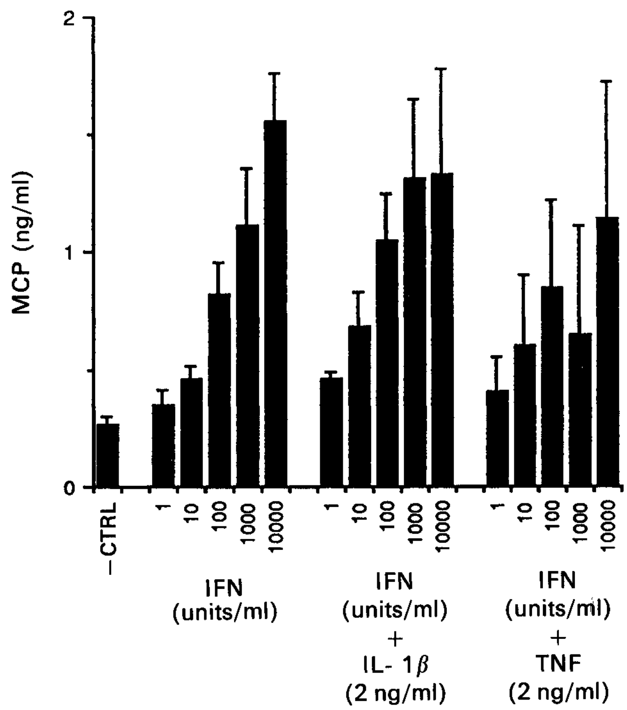

FIG. 2. Dose dependent monocyte derived antigenic MCP-1 expression post-stimulation by IFN-γ, either alone or in combination with IL-1β, TNF-α, as determined by ELISA. *Indicates $p < 0.05$ compared with control monocytes at comparable time point (experimental $n = 7$).

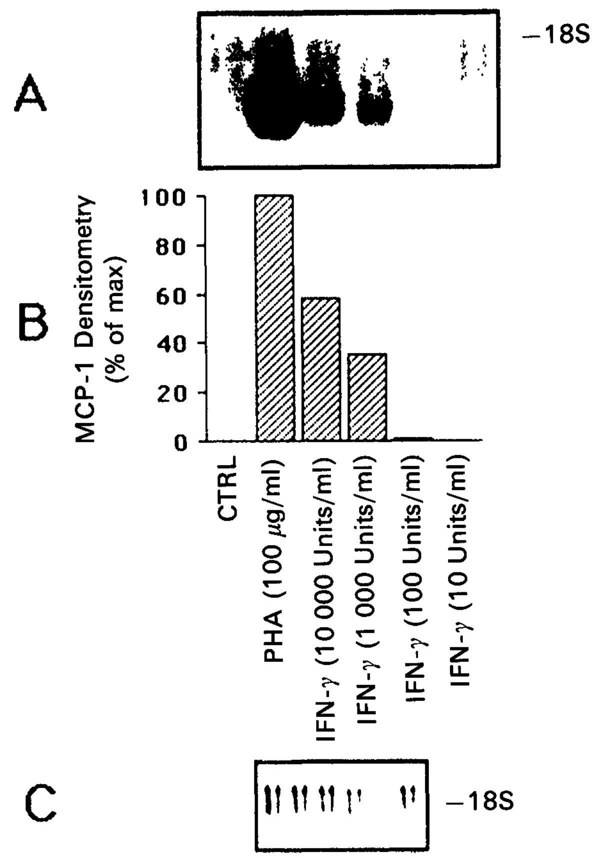

FIG. 3. Northern blot of monocyte derived MCP-1 post-stimulation by varying doses of IFN-γ, as indicated. Cells were incubated in conditioned media for 24 h. (A) Representative Northern blot of monocytes. (B) Results of densitometry of Northern blot. (C) Corresponding 18S and 28S rRNA for the Northern blot in panel A, demonstrating equivalent loading of RNA (experimental $n = 2$).

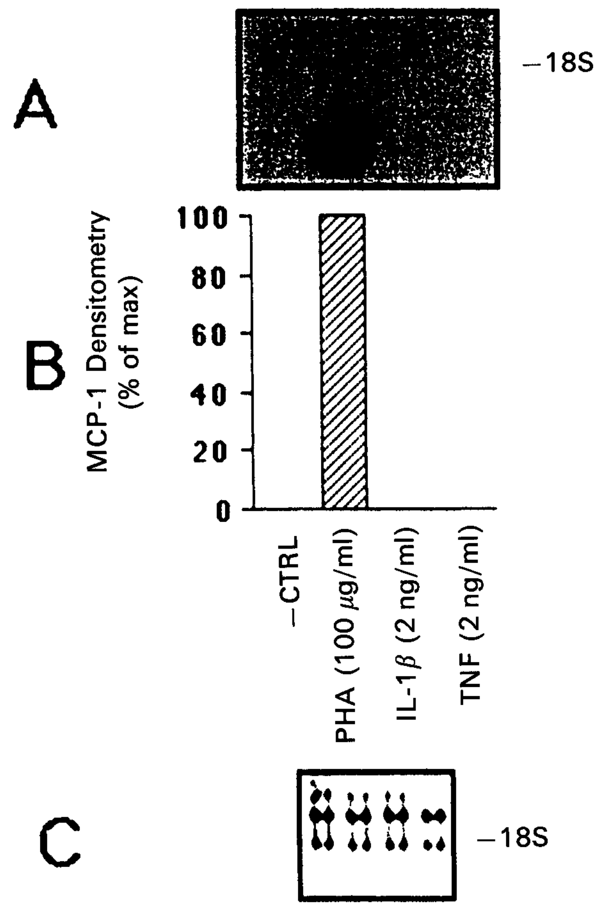

FIG. 4. Northern blot of monocyte derived MCP-1 mRNA stimulated by PHA, IL-1$\beta$, or TNF-$\alpha$, as indicated. Cells were incubated in conditioned media for 24 h. (A) Representative Northern blot of monocytes. (B) Results of densitometry of Northern blot. (C) Corresponding 18S and 28S rRNA for the Northern blot in panel A, demonstrating equivalent loading of RNA (experimental $n = 2$).

stimulation using PHA (100 $\mu$g/ml). There was no apparent expression of MCP-1 mRNA following stimulation with IL-1$\beta$ (2 ng/ml) or TNF-$\alpha$ (2 ng/ml) as shown in Fig. 4. MCP-1 mRNA expression was also not detectable following stimulation with IL-4 (10 ng/ml, data not shown).

### Discussion

MCP-1 is a secreted 76-amino acid peptide with potent monocyte chemotactic and activating properties.[6] MCP-1 is a member of the C–C chemotactic cytokine supergene family of pro-inflammatory peptides which includes RANTES,[11] MIP-1,[12] MCP-2[13] and MCP-3.[13] A variety of cultured cells are able to synthesize MCP-1, including endothelial cells,[14,15] fibroblasts,[16] vascular smooth muscle cells,[17] keratinocytes,[18] synovial cells,[19] pulmonary alveolar type II-like cells,[20] various tumour cells,[21,22] and peripheral blood mononuclear cells in the presence of serum.[4,23,24] A serine/threonine protein kinase signalling pathway is thought to mediate the effects of MCP-1 induced monocyte chemotaxis.[25]

It has been suggested that the inappropriate expression of MCP-1 by immune and non-immune cells may be associated with the pathophysiology of various diseases mediated by monocytes/macrophages.[26] Investigators have found MCP-1 protein and mRNA in the abnormal joints of patients with rheumatoid arthritis,[2] in lung tissue from patients with idiopathic pulmonary fibrosis,[3] and in atheromatous plaques from patients with atherosclerosis.[1,27,28] Increased antigenic MCP-1 was demonstrated in supernatants of cultured pulmonary alveolar macrophages from rats with immune complex-mediated acute lung injury,[29] and in bronchoalveolar lavage fluid from rats with glucan induced pulmonary granulomatosis.[30] Of interest, monocytes/macrophages were identified as expressing MCP-1 protein or mRNA in each of the above human or experimental diseases. The production of monocyte/macrophage-derived MCP-1 may be an important factor in the initiation or maintenance of these inflammatory states. However, the regulation of monocyte/macrophage-derived MCP-1 is as yet unclear.

In this study the authors attempted to learn under what conditions monocytes cultured in serum free media express MCP-1 mRNA and protein. Although monocyte derived MCP-1 expression was induced by PHA, it was found that monocytes did not generate MCP-1 in response to IL-1$\beta$ or TNF-$\alpha$. In contrast, other investigators have found that IL-1$\beta$ and TNF-$\alpha$ both stimulate MCP-1 production from endothelial cells,[15] fibroblasts,[31] epithelial cells,[5] vascular smooth muscle cells[17] and mesangial cells.[32] IL-4, an immunoregulatory peptide produced primarily by the $T_H2$ subset of T lymphocytes[33] also did not induce the production of MCP-1. It was found, however, that stimulation with IFN-$\gamma$ resulted in a dose dependent increase in MCP-1 mRNA and protein. Simultaneous stimulation with IFN-$\gamma$ and IL-1$\beta$ or TNF-$\alpha$ did not alter the response to IFN-$\gamma$ alone.

IFN-$\gamma$ has previously been identified as a potent regulator of monocyte and macrophage functions including enhanced monocyte MHC class and II antigen expression,[34] increased antimicrobial activity,[35] and stimulation of the respiratory burst.[36] IFN-$\gamma$ is largely a product of the $T_H1$ cell type $CD4^+$ T lymphocytes.[37,38] This subset of helper T cells is predominately involved in delayed type hypersensitivity responses (DTH).[38] IFN-$\gamma$ has been found in many DTH type diseases such as sarcoidosis,[39] tuberculoid lesions of leprosy[40] and experimental *Schistosoma mansoni* infection.[41] Thus, IFN-$\gamma$ is likely to be present in several types of inflammatory conditions and may be capable of stimulating monocyte/macrophage derived MCP-1 production *in vivo*. It was noted that the IFN-$\gamma$ stimulated MCP-1 production by monocytes was modest compared with that of the nonspecific monocyte activator, PHA. It is possible that factors other than IFN-γ may stimulate MCP-1 production by monocytes/macrophages or may act synergistically with IFN-γ. These factors may arise from T lymphocytes or from other cells in the inflammatory milieu. However, the identity of such factors is as yet unknown.

In summary, a dose dependent increase in monocyte derived MCP-1 production was observed following stimulation with IFN-γ under serum free conditions. Monocytes did not produce MCP-1 in response to stimulation with IL-1β, TNF-α or IL-4. In addition, no change in MCP-1 production was found when cells were stimulated with IFN-γ in the presence of IL-1β or TNF-α compared with that seen with IFN-γ alone. These findings suggest that the generation of monocyte derived MCP-1 production is signal specific. It is concluded that IFN-γ, primarily a product of $T_H1$ lymphocytes, is a primary stimulus for the production of monocyte derived MCP-1.

### References

1. Nelken NA, Coughlin SR, Gordon D, Wilcox JN. Monocyte chemoattractant protein-1 in human atheromatous plaques. *J Clin Invest* 1991; **88:** 1121–1127.
2. Koch AE, Kunkel SL, Harlow LA, *et al.* Enhanced production of monocyte chemoattractant protein-1 in rheumatoid arthritis. *J Clin Invest* 1992; **90:** 772–779.
3. Antoniades HN, Neville-Golden J, Galanopoulos T, Kradin RL, Valente AJ, Graves DT. Expression of monocyte chemoattractant protein 1 mRNA in human idiopathic pulmonary fibrosis. *Proc Natl Acad Sci USA* 1992; **89:** 5371–5375.
4. Colotta F, Borre A, Wang JM, *et al.* Expression of a monocyte chemotactic cytokine by human mononuclear phagocytes. *J Immunol* 1992; **148:** 760–765.
5. Kunkel SL, Standiford T, Kasahara K, Strieter RM. Stimulus specific induction of monocyte chemotactic protein-1 (MCP-1) gene expression. In: Westwick J, Lindley IJD, Kunkel SL eds., *Chemotactic Cytokines: Biology of the Inflammatory Peptide Supergene Family*. New York and London: Plenum Press, 1991; 65–71.
6. Yoshimura T, Robinson EA, Tanaka S, Appella E, Leonard EJ. Purification and amino acid analysis of two human monocyte chemoattractants produced by phytohemagglutinin-stimulated human blood mononuclear leukocytes. *J Immunol* 1989; **142:** 1956–1962.
7. Chirgwin JM, Przybyca AE, MacDonald RJ, Rutter WJ. Isolation of biologically active ribonucleic acid from sources rich in ribonuclease. *Biochemistry* 1979; **18:** 5294.
8. Jonas E, Sargent TD, Davis IB. Epidermal keratin-gene expressed in embryos of *Xenopus laevis*. *Proc Natl Acad Sci USA* 1985; **82:** 5413.
9. Furuntani Y, Nomura H, Notake M, *et al.* Cloning and sequencing of the cDNA for human monocyte chemotactic and activating factor (MCAF). *Biochem Biophys Res Comm* 1989; **159:** 249.
10. Evanoff HL, Burdick MD, Moore SA, Kunkel SL, Strieter RM. A sensitive ELISA for the detection of human monocyte chemoattractant protein-1 (MCP-1). *Immun Invest* 1992; **21:** 39–45.
11. Schall TJ, Bacon K, Toy KJ, Goeddel DV. Selective attraction of monocytes and T lymphocytes of the memory phenotype by cytokine RANTES. *Nature* 1990; **347:** 669–671.
12. Wolpe SD, Cerami A. Macrophage inflammatory proteins 1 and 2: members of a novel superfamily of cytokines. *FASEB J* 1989; **3:** 2565–2573.
13. Van Damme J, Proost P, Lenaerts J-P, Opdenakker. Structural and functional identification of two human, tumor-derived monocyte chemotactic proteins (MCP-2 and MCP-3) belonging to the chemokine family. *J Exp Med* 1992; **176:** 59–65.
14. Rollins BJ, Yoshimura T, Leonard EJ, Pober JS. Cytokine-activated human endothelial cells synthesize and secrete a monocyte chemoattractant, MCP-1/JE. *Am J Pathol* 1990; **136:** 1229–1233.
15. Sica A, Wang JM, Colotta F, *et al.* Monocyte chemotactic and activating factor gene expression induced in endothelial cells by IL-1 and tumor necrosis factor. *J Immunol* 1990; **144:** 3034–3038.
16. Yoshimura T, Leonard EJ. Secretion by human fibroblasts of monocyte chemoattractant protein-1, the product of gene JE. *J Immunol* 1990; **144:** 2377–2383.
17. Wang JM, Sica A, Peri G, *et al.* Expression of monocyte chemotactic protein and interleukin-8 by cytokine-activated human vascular smooth muscle cells. *Arterioscler Thromb* 1991; **11:** 1166–1174.
18. Barker JNW, Jones ML, Swenson CL, *et al.* Monocyte chemotaxis and activating factor production by keratinocytes in response to IFN-γ. *J Immunol* 1991; **146:** 1192–1197.
19. Hachicha M, Rathanaswami P, Schall TJ, McColl SR. Production of monocyte chemotactic protein-1 in human type B synoviocytes. *Arthritis Rheum* 1993; **36:** 26–34.
20. Standiford TJ, Kunkel SL, Phan SH, Rollins BJ, Strieter RM. Alveolar macrophage-derived cytokines induce monocyte chemoattractant protein-1 expression from human pulmonary type II-like epithelial cells. *J Biol Chem* 1991; **266:** 9912–9918.
21. Zachariae COC, Thestrup-Petersen K, Matsushima K. Expression and secretion of leukocyte chemotactic cytokines by normal human melanocytes and melanoma cells. *J Invest Dermatol* 1991; **97:** 593–599.
22. Jiang Y, Valente AJ, Williamson MJ, Zhang L, Graves DT. Post-translational modification of a monocyte-specific chemoattractant synthesized by glioma, osteosarcoma, and vascular smooth muscle cells. *J Biol Chem* 1990; **265:** 18318–18321.
23. Yoshimura T, Takeya M, Takahahi K, Kuratsu J-I, Leonard EJ. Production and characterization of mouse monoclonal antibodies against human monocyte chemoattractant protein-1. *J Immunol* 1991; **147:** 2229–2233.
24. Brach MA, Gruss H-J, Riedel D, Asano Y, De Vos S, Herrmann F. Effect of antiinflammatory agents on synthesis of MCP-1/JE transcripts by human blood monocytes. *Mol Pharmacol* 1992; **42:** 63–68.
25. Sozzani S, Luini W, Molino M, *et al.* The signal transduction pathway involved in the migration induced by a monocyte chemotactic cytokine. *J Immunol* 1991; **147:** 2215–2221.
26. Rollins BJ. "Oh, No. Not another cytokine."— MCP-1 and respiratory disease. *Am J Respir Cell Mol Biol* 1992; **7:** 126–127.
27. Ylä-Herttuala S, Lipton BA, Rosenfeld ME, *et al.* Expression of monocyte chemoattractant protein-1 in macrophage-rich areas of human and rabbit atherosclerotic lesions. *Proc Natl Acad Sci USA* 1991; **88:** 5252–5256.
28. Koch ALE, Kunkel SL, Pearce WH, *et al.* Enhanced production of the chemotactic cytokines interleukin-8 and monocyte chemoattractant protein-1 in human abdominal aortic aneurysms. *Am J Pathol* 1993; **142:** 1423–1431.
29. Brieland JK, Jones ML, Clarke SJ, Baker JB, Warren JS, Fantone JC. Effect of acute inflammatory lung injury on the expression of monocyte chemoattractant protein-1 (MCP-1) in rat pulmonary alveolar macrophages. *Am J Respir Cell Mol Biol* 1992; **7:** 134–139.
30. Jones ML, Warren JS. Monocyte chemoattractant protein 1 in a rat model of pulmonary granulomatosis. *Lab Invest* 1992; **66:** 498–503.
31. Rolfe MW, Kunkel SL, Standiford TJ, *et al.* Expression and regulation of human pulmonary fibroblast-derived monocyte chemotactic peptide-1. *Am J Physiol* 1992; **263:** L536–L545.
32. Rovin BH, Yoshimura T, Tan L. Cytokine-induced production of monocytes chemoattractant protein-1 by cultured human mesangial cells. *J Immunol* 1992; **148:** 2148–2153.
33. Paul WE. Interleukin-4: a prototypic immunoregulatory lymphokine. *Blood* 1991; **77:** 1859–1870.
34. Celada A, Maki RA. IFN-γ induces the expression of the genes for the MHC Class II I-Aβ and tumor necrosis factor through a protein kinase C-independent pathway. *J Immunol* 1991; **146:** 114–120.
35. Nathan CF, Murray HW, Wiebe ME, Rubin BY. Identification of interferon-γ as the lymphokine that activates human macrophage oxidative metabolism and antimicrobial activity. *J Exp Med* 1983; **158:** 670–689.
36. Abbas AK, Lichtman AH, Pober JS. *Cellular and Molecular Immunology*. Philadelphia, Pennsylvania: W.B. Saunders Company, 1991.
37. Mosmann TR, Cherwinski H, Bond MW, Giedlin MA, Coffman RL. Two types of murine helper T cell clone. I. Definition according to profiles of lymphokine activities and secreted proteins. *J Immunol* 1986; **136:** 2348–2357.
38. Salgame P, Abrams JS, Clayberger Cl, *et al.* Differing lymphokine profiles of functional subsets of human CD4 and CD8 T cell clones. *Science* 1991; **254:** 279–282.
39. Robinson B, McLemore T, Crystal RG. Gamma interferon is spontaneously released by alveolar macrophages and T lymphocytes in patients with pulmonary sarcoidosis. *J Clin Invest* 1985; **75:** 1488–1495.
40. Yamamura M, Uyemura K, Deans RJ, *et al.* Defining protective responses to pathogens: cytokine profiles in leprosy lesions. *Science* 1991; **254:** 277–282.
41. Chensue SW, Terebuh PD, Warmington KS, *et al.* Role of IL-4 and IFN-γ in *Schistosoma mansoni* egg-induced hypersensitivity granuloma formation. *J Immunol* 1992; **148:** 900–906.

ACKNOWLEDGEMENTS. This research was supported in part by National Institutes of Health grants 1P50HL46487, HL02401, HL31693, HL35276, HL02701 and DK38149. Dr Strieter is an RJR Nabisco Research Scholar.

**Received 28 September 1993; accepted in revised form 16 November 1993**
